# Diminishing pain stigma: patient perceptions of encounters with interprofessional teams in biopsychosocial pain rehabilitation

**DOI:** 10.1080/07853890.2022.2124447

**Published:** 2022-09-20

**Authors:** Gudrun S. Battin, Grace I. Romsland, Bjørg Christiansen

**Affiliations:** a Department of Nursing and Health Promotion, Oslo Metropolitan University, Oslo, Norway; b Department of Public Health, University of Stavanger, Stavanger, Norway; c Sunnaas Rehabilitation Hospital, Nesodden, Norway

**Keywords:** Rehabilitation, chronic pain, stigma, interprofessional collaboration, teamwork, biopsychosocial, ethnography

## Abstract

**Purpose:**

To explore how patients in biopsychosocial pain rehabilitation perceive encounters with interprofessional teams. The focus of this article is to explore how interactions can perpetuate or diminish chronic pain stigma.

**Material and methods:**

An ethnographic approach was applied to the study. Participant observation of interprofessional encounters and clinical encounters in a pain rehabilitation ward was undertaken in 2016 (19 weeks). Interviews with 12 professionals and seven patients were conducted. Data were analysed in an abductive process using thematic analysis.

**Results:**

The patients perceived their encounters with interprofessional teams as supportive, with implications for pain stigma. This is presented as two themes: (1) being seen as credible, involving patients being believed in and a concept of being overactive at the expense of their own health, and (2) being helped to see their situation in a new light, which involves enthusiasm about changing and challenging views in a process with professionals who were supportive and united across professions.

**Conclusion:**

Interprofessional biopsychosocial pain rehabilitation may be an intervention that can diminish internalised stigma in patients suffering from chronic pain. The study contributes to increased understanding of patient perceptions of positive encounters with professionals during a learning process in rehabilitation and of the imbued influence on power relations. This appears to be fundamental to diminishing pain stigma, as the occurrence of stigma is dependent on differences in power.

**KEY MESSAGES**
- Interprofessional biopsychosocial pain rehabilitation can be an intervention for diminishing internalised pain stigma in patients.
- Knowledge on how encounters with professionals induce personal learning processes among people with chronic pain.

## Introduction

In modern society, people with chronic pain suffer from stigmatising attitudes [1,2]. Health care professionals such as physiotherapists, nurses, and medical doctors have been found to have attitudes consistent with social stigma attached to pain [1] which can reduce quality of life and patient trust in receiving appropriate treatment from the health care system [1,3]. The most common stigmatising attitudes are especially prominent when no clear explanation for the pain can be found. Such attitudes among professionals and the general population include suspecting deception, attributing less pain, feeling less sympathy, disliking patients, and being less inclined to help than when the pain is acute or when tissue pathology is found [1,4].

Patients with chronic pain have been found to invest much work and energy in being perceived as credible by professionals [5]. At the same time, internalised stigma in patients with chronic pain does not seem to be well known in clinical practice [1,2]. Despite the challenging outset for patients, biopsychosocial pain rehabilitation can reduce pain and disability in patients suffering from chronic pain using a multimodal interprofessional approach [6,7]. The purpose of this study was to explore how patients in biopsychosocial pain rehabilitation perceive encounters with interprofessional teams. The focus of this article is on how interactions may perpetuate or diminish stigma.

### Background

#### The stigma concept

For the purpose of this study, we find Link and Phelan’s [8, p.367] concept of stigma useful. They found stigma to unfold in power situations where several interrelated components co-occur. The first component is labelling and distinguishing between human differences. The second is creating negative stereotypes, when dominant cultural beliefs associate labelled people with undesirable characteristics and a separation between “us” and “them” can occur. The last two components are status loss and discrimination. This conceptualisation expands on Goffman’s [9] view on stigma as occurring in the relationship between an attribute and a stereotype. In this article the view of stigma as the interrelation between social components provides a tool to explore how interactions and accounts from professionals and patients can perpetuate or diminish stigma. Also, the dependence on differences in power is relevant in the exploration of interactions between patients and interprofessional teams; those who stigmatise need to have a social, cultural, political or economic power to make their perceptions about a group entail serious discriminatory consequences [8,10].

#### Internalised stigma in patients with chronic pain

Internalising stigma is not a necessary consequence of social stigma [2,3]. Some people are unaware of stigma, some are unmotivated by it or recognise the stigmatising attitudes as unjust and set out to change them [3]. However, Waugh et al. [2] found that 38% of people living with chronic pain internalised the stigma. One reason why patients tend to internalise the stigma may be a lack of any clear explanation for the pain, making them insecure about the nature and reality of their own pain [1].

Research has found multiple consequences of social stigmatising attitudes towards people with chronic pain. Internalisation of pain stigma is associated with low self-esteem and low self-efficacy [2]. Perceptions of unfairness about their own, low status compared with others in similar situations can lead to depression, anxiety, prolonged work disability and poorer rehabilitation outcomes in people with chronic pain [1].

#### Biopsychosocial pain rehabilitation and diminishing stigma

Interprofessional biopsychosocial pain rehabilitation is often defined as rehabilitation programs delivered by a team of at least two separate professions which communicate extensively about patient management, targeting at least two of the following aspects to meet the complexity of chronic pain: physical, psychological, social and work-related factors. The service can be provided in rehabilitation centres, pain clinics or outpatient settings [6,11–13]. An increasing knowledge base shows that biopsychosocial pain rehabilitation has an effect on pain and disability in patients with chronic pain [6,7,14]. However, the design of these rehabilitation programs varies widely, with scarce knowledge to inform what critical components and mechanisms are in play to entail success [6]. In interprofessional collaboration, the worldviews of professions may be more biomedical or more biopsychosocial [14], and these will have different implications for how patients with chronic pain are met and treated. Still, if the differences are used to complement each other, there is potential to provide optimal services for patients [15,16].

According to Wade [17], learning is a fundamental patient process in rehabilitation to reach the goal of reducing limitations on activities in the patient’s life. “Learning” can be viewed as a change in capability or disposition which is relatively permanent [18]. At the individual level of health education (as empowerment), learning is about strengthening the capacity to control one’s own health. Patient education can be included in the concept of health education in the empowerment model of health promotion [18,19]. While patient education has frequently been studied as a planned activity, earlier research has found it to be both a formal and informal activity between professionals and patients [20–23]. The informal can be characterised as teaching activities which are intentional while not highly structured [20]. The activities can include coaching and mentoring [20], providing instructions, giving information, asking questions, demonstrating correct performance or giving explanations [22].

Professionals across professions in health care have been found to have stigmatising attitudes towards patients with chronic pain [1,4]. Increased knowledge about a biopsychosocial explanation for chronic pain and pain management strategies is suggested to lead to decreased stigmatising attitudes [1,24]. Alongside reducing health care professionals’ stigmatising attitudes towards patients, empowering patients has been suggested as a central way to counteract internalised stigma. However, more research on factors that contribute to an empowering process in the face of pain stigma is needed to inform professional practice [2,3]. Thus, the field of interprofessional biopsychosocial pain rehabilitation can be a place to gain needed knowledge.

## Materials and methods

### Design

The study is based on findings from an overall qualitative study using an ethnographic approach to explore social processes characterising interprofessional collaboration in pain rehabilitation [25,26]. The use of participant observation combined with semi-structured interviews enabled us to explore actions and accounts of interprofessional teams and their patients [27], where patient and professional perceptions of the social processes enriched the understanding of the observed actions. In the present article, we focus on themes developed with special attention to data from patient interviews and observed encounters between professionals and patients.

### Setting and participants

The setting for the study was a biopsychosocial pain rehabilitation in-patient ward in a hospital in Norway. Overall, 19 professionals and 26 patients participated in the observation and interviews in the study. Two intertwined interprofessional teams provided the rehabilitation program, in a close-knit team setting [28]. We selected the setting to reflect complex interprofessional collaboration with patients suffering from chronic pain. Biopsychosocial pain rehabilitation is characterised by a collaborative design with multiple professions involved to meet the multiple dimensions of chronic pain [6]. We chose one single ward to facilitate in-depth investigation of the social processes [25].

The teams consisted of registered nurses, physiotherapists, occupational therapists, psychologists, medical doctors, and social workers. The two teams in the ward were intertwined, as some shared office space across teams and some held functions in both teams. Their approach had a cognitive focus combined with patient education about topics such as pain physiology, and physical activities such as strength training. Adjusting medication or introducing invasive pain treatment was not part of the program. One team provided an individual program with more use of one-on-one appointments with each profession. The other team facilitated a group-based program with more use of group education, counselling, and training sessions. This team also had some individual admissions.

The patients suffered from chronic pain with basis in a wide range of causes such as physical trauma or hypermobility syndrome or with no biomedical findings explaining the pain. Diagnosis was not included in the criteria for admission. To qualify for the rehabilitation programme, patients had to have complex pain conditions with a severity that led to significant dysfunction in daily activities and reduced quality of life. They had to have completed investigation into the cause of the pain. Moreover, patients had to be evaluated as motivated for a biopsychosocial approach and in need of interprofessional rehabilitation. The patients stayed in the hospital unit for approximately four periods lasting from one to four weeks during a time span of one year.

### Data collection

#### Observation

The first author conducted participant observation between February and June 2016 on 40 unique days of approximately 5 h. The observational role varied from more to less participation, which is common in participant observation [25]. The first author was welcomed into the field by both patients and professionals with friendly talk and invitations to observe. The easy access may be due to the first author having several characteristics similar to the patients and professionals, which according to Hammersley and Atkinson [25] affects relationships and thus access to information in the field. These characteristics included the observer being a woman and a native Norwegian like most of the patients and professionals, having a background as a registered nurse and wearing casual clothing.

The first author observed encounters between patients and professionals from all the six professions involved. In encounters between patients and professionals, attention was given to the actions and accounts of both parties. These encounters could take the form of counselling (seven occasions), discharge meetings (four occasions), patient education (12 occasions), physical activities (four occasions) and informal encounters. Interprofessional encounters such as team meetings, informal conversations and written reports were observed. The focus of the observations intentionally interchanged between aspects such as the content of stories, the non-verbal social atmosphere or the words used. For example, in a meeting between an occupational therapist and patient, focus was placed on the exact words used because the conversation appeared to be substantial and to flow well. During group patient education sessions, where professionals could hold a monologue for some time, focus might be placed on the patients’ changing non-verbal signals of being concentrated, bored or in pain. Field notes (total length of 51,010 words) were written during observation or as soon as possible afterwards.

#### Interviews

During the observation phase, the first author conducted semi-structured individual interviews (19) with patients (7) and professionals (12). Patient interviews had an average length of 41 min and interviews with professionals an average length of 54 min. Interview guides with open-ended questions were used. Examples of questions in the interview guide for patients include “What was it like to meet the professionals who work here?”, “Can you tell me about an experience of meeting the professionals here that has been especially rewarding for you?”, “Can you tell me about an experience of meeting the professionals here that has been particularly challenging?” and “What is your experience of telling the professionals about your pain?” The interviews were undertaken to obtain accounts to broaden the understanding of the observed social processes [29]. None of the individuals who were asked to be interviewed refused. The interviews were recorded and then transcribed verbatim.

We selected the patients with the aim of representing a diversity of perspectives based on pain background, gender, experiences from group-based or individual admissions in the unit, and on being perceived by professionals to vary in their perspectives on the rehabilitation process. A nurse from one of the observed teams helped recruit potential patient participants. Three of the patients interviewed were also observed. Among the 12 professionals who were interviewed, two participants from each of the six professions were represented. They were recruited by the first author during fieldwork in the hospital unit, with the aim of obtaining varying accounts from all professions. Professionals were selected based on convenience, when there for instance were only two employed psychologists, or on experience or distinct perspectives needed to expand the data.

### Ethical considerations

The hospital’s data protection officials approved the study on behalf of the Norwegian Data Protection Authority, which cooperates with the Regional Committee for Medical and Health Research Ethics. All participants received written information about the study and signed an informed consent letter. At the start of the fieldwork, the first author presented herself and informed the two teams about the study in interprofessional meetings. This was also done before observing groups of patients. Before observing one-on-one encounters between patients and professionals, an agreement was made with the professional, who then asked and informed the patient. If the patient consented, the first author informed the patient about the study at the start of the observation. The first author was present for dialog with all participants during the fieldwork. Interview settings were selected to ensure confidentiality, and sensitive information was deleted in publications.

### Data analysis

We used thematic analysis [30,31] to develop themes from the data, combined with a constructionist framing [25,26] and an abductive orientation [32] which urged a back-and-forth movement between literature and data leading to selection of a theoretical framework that could bring the most out of the data. The authors discussed the emerging analysis iteratively throughout the research process. HyperRESEARCH software was used to systematically manage the data [33].

Analysis began during fieldwork with writing notes about patterns and ideas. Influenced by these notes, the first author generated an unrefined map of codes and themes across the data [30]. The starting point for the themes presented in the findings of this article was a pattern of patients frequently talking about how other people in society and the health care system misinterpreted them, while they appeared to be very satisfied with the teams and their rehabilitation program. We wished to explore the social meaning of the diversity in these accounts and interactions. We further developed the themes through continued reading of related data and engaging the data with a wide range of literature sources. We found Link & Phelan’s [8] stigma concept and the concept of patient education as empowerment [18,19] especially fruitful in identifying social meaning in the patients’ perceptions of encounters with interprofessional teams. We landed on the final themes at a fairly semantic level to discern the concrete perceptions and interactions of the participants, while in the discussion the analysis was brought further into the constructionist frame by theorising the latent significance concerning pain stigma.

### Rigour

We addressed the trustworthiness of the study in several ways, which according to Lincoln [34] can be divided into credibility, dependability, transferability, and confirmability. A reflective journal was written about thoughts, ideas and plans during data collection and analysis. Peer debriefing was applied when the first author regularly discussed the process with the co-authors during data collection and analysis. These actions can strengthen the credibility of the study [35]. Prolonged engagement with the participants during observation to provide rich data was also a way of strengthening credibility [36].

Journaling also strengthens the dependability of the research process [36]. In addition, some of the field notes were methodological notes about who to interview and what to observe further in the fieldwork that would strengthen dependability. The field notes included detailed information about factors such as the surroundings, atmosphere, and the people present to bring about a vivid picture of the events. The interview questions were peer-reviewed with the co-authors in advance in order to gather thick descriptions. The questions were open ended. The interviews were conducted with an awareness of obtaining detailed responses with concrete examples. The actions of building thick descriptions and journaling facilitated transferability [35], since they enable the reader to determine their transferability to other times, settings and people [36].

The participating professionals and patients were sampled in such a way as to represent a variety of perspectives and broaden the understanding of the phenomena, which reinforces confirmability. The concept of confirmability can be explained as the extent of the researchers’ prior understanding of the phenomenon shaping what is found, where the researcher should aim to use data gathering procedures that confirm findings as also shaped by participants [36]. Comparing data from people with differing viewpoints serves as a form of source triangulation. In collecting data, we combined observation with interviews, which allowed checking for consistency across different data collection methods. This is a way of triangulating methods. These forms of triangulation can strengthen the confirmability of the study [35].

## Results

We identified two themes showing the patients’ perceptions of their encounters with interprofessional pain rehabilitation teams, with implications for pain stigma: (1) to be seen as credible, and (2) being helped to see their situation in a new light.

### To be seen as credible

Tom was sitting in his room when I came to talk to him. He had struggled with chronic pain stealing his time and energy for years. He experienced a wide gap between his current situation and his longing to spend time with family and friends and do sports. Exhausted from pain after performing chores such as vacuum cleaning, he would have to lie on the coach for the rest of the day. He felt that he had to tiptoe around people who wrongly assumed him to be in good health and expected him to behave in ways he could not. The rehabilitation ward was for him a free space where he could finally relax in a social dimension, although the rehabilitation was hard work and he went through one disappointment after the other on his journey.

[…] you feel safe when you come here. You can relax, you can be yourself. You don’t have to tiptoe and sort of: ‘is anyone looking down on me for parking in the handicap space? It… he looks healthy, so he doesn’t need to park there!’ Even though I’m allowed to. (Patient, interview)

The patients feared others misinterpreted them as lazy or slackers for working fewer hours or not at all or for not being there for their family and friends because they needed a lot of rest. These others did not see how the patients gave all they had, did too much, wanted to achieve too much and did not think enough about their own needs. This was expressed in interviews, in patient education sessions and in one-to-one meetings between professionals and patients.

Through meeting the professionals, the patients found they were finally seen as credible, which was followed by a new or strengthened positive self-understanding. Especially the concept of “overactivity” appeared to make them feel understood and credible by the professionals. This concept was about them exerting themselves in the wrong way, often driven by the fear of being negatively and stereotypically misinterpreted. This exertion led to more pain and exhaustion. All the patients talked about this in the interviews, and five of them also linked it to their perception of the professionals understanding them in the right way:

There’s a fear, it’s … a protection mechanism that makes you exert yourself completely wrong and do even more. So, all those things, the inner things, let go [through rehabilitation]. I was left with only the real pain. And the fear was somehow gone. (Patient, interview)

Trying to protect themselves from stereotypical views, at the expense of their own health, implies that the patients were aware of and cared about negative perceptions in society. They appeared to be balancing between believing in the negative perceptions and taking care of their own needs. The need to be careful not to be overactive on good days resonated well with the patients. They needed to work on overactivity so that they could live a better life with less pain and participate in everyday life. When passing on this view to the patients, the professionals referred to how it was a widespread problem among people with chronic pain, and visualised their message using hand drawings and metaphors. Such interactions were observed in one-to-one meetings and patient education sessions, and can be illustrated by a meeting between an occupational therapist and a patient:

Patient: ‘I have a long list of things I want to do on a good day. Yesterday, I talked to the physiotherapist about doing as little on a good day as on a bad day. To gain energy … For the past six months, the normal days have been completely gone!’ Occupational therapist: ‘Many with long-term pain talk about such a pattern.’ She draws a line with large wave crests and deep troughs on a sheet of paper. ‘It alternates between the good days where a lot is done, followed by very bad days.’ The patient recognizes the pattern and says: ‘In those troughs, the bad days, I just lie all day.’ The occupational therapist draws a line with smaller waves that run through the middle of the big waves and says: ‘It’s important to live a life which is more like this.’ Patient: ‘I have to slow down on the good days.’ (Field notes, meeting between occupational therapist and patient)

The patients were taught about the problem of having too many rules for not participating in everyday life in order to avoid pain, and about balancing their activities so as not to be overactive. Still, through interactions between patients and professionals concerning the right balance between not being overactive and not having pain rules, most patients expressed a perception of being understood as first and foremost overactive.

In five of the interviews, patients talked about how they did not tend to whine and complain about pain. They avoided talking about illness because they believed it might become worse. They did not want to appear to others as sick or be a burden by making people feel sorry for them. The pain was not what they wanted to talk about with the professionals.

I'm not the kind of person who talks that much about illness. Eh … in general. So … I think it’s too much. I think it … becomes negative somehow. Instead, I want to focus on the nice weather outside and that I want to go for a walk, because it … does something more to you than to… [focus on illness] (laughs a little) yes. (Interview, patient)

In three interviews, patients expressed relief at being believed when they talked about their pain with the professionals in the rehabilitation unit:

The first time [he told the rehabilitation professionals about his pain] it was … uncomfortable … Because I had experiences from the health care system, I was not completely believed by everyone [he takes a deep breath] … And I was just a like ball being thrown around in a system, back and forth in the hospitals for many years. New examinations and . yes … But when I came here and … feeling safe after the first conversation … You can drop your shoulders and: ‘Oh, finally! Finally, there’s someone who has … believes in me.’ (Interview, patient)

Only to a small extent was pain observed to be directly discussed between patients and professionals in pain rehabilitation, although it could be a topic in their first encounters. Rather, several patients expressed a wish to communicate with the professionals about what affected the pain and what could be done. There appeared to be a widespread wish to receive help with seeing their demanding life with chronic pain in a new light.

### Being helped to see their situation in a new light

There was widespread enthusiasm among the patients about the biopsychosocial approach. According to talks between patients and accounts given in six of the interviews, many appeared to have strong positive emotions connected to their new insights and how the professionals in the rehabilitation program were outstanding and could be trusted more than elsewhere. In interviews, such accounts dealt with being privileged to receive such good treatment or with how the professionals’ input and tools had helped them to see their situation in a new light. Sarah, a woman in her forties who had suffered from pain all her life due to hypermobility syndrome, expressed great enthusiasm about how her views had been changed through a process in which she needed time alone to digest the information:

When he drew it up and, I didn’t get it right away, but when I read it afterwards in the evenings and sort of looked at it a bit… because there’s a lot of information to take in and you become like, wow. And then there’s another worldview … I don’t know about … I will use a word called paradigm, it’s a small paradigm shift in your own thinking! Because you have been there, and you feel like the pain has had catastrophic consequences. Been a lot of illness, pain and all. And you’re a patient and you’re almost a victim and … and then suddenly: yes, but we can separate something away here! (Interview, patient)

The accounts from patients could deal with experiencing the professionals as fellow human beings who listened to what they said while giving clear advice. Five patients expressed in the interviews that the professionals collaboratively acted on the information they provided. Sarah was very pleased with how she had been met interprofessionally:

And you meet real people before you meet the professional […] And what’s really surprising is that when you say something, they actually listen to you. And you get good answers back. And they come back to you afterwards and say: ‘Sarah, you said that, now we have thought a bit and discussed in the team, because they have such interdisciplinary meetings, and we have found out … how does that sound to you?’ And I think that’s good and professional, I like that. (Interview, patient)

Two patients said they had heard different stories from other patients in the unit who had negative perceptions of the help given. The negative perceptions were explained as being due to prior experiences with the health care system or to not understanding what the rehabilitation program was about.

I'm more naïve; this [rehabilitation] worked really well! Or it may not be naive, but I think the experiences from when we have met the health service … I have had them [health care professionals] in my life for a short period, while they [other patients] have often had them a long while. So that’s why I also think that I see it with different eyes, and when it works, it works so well because … I don’t have that negative ballast with me. (Interview, patient)

The patients talked about how hard it was to trust whether it was safe to do as professionals said due to earlier experiences of how professionals approached chronic pain in different ways and gave contrary information or advice concerning the pain. This is illustrated by the following observed talk between patients about their earlier experiences with professionals elsewhere:

One patient said: ‘They work in such different ways. Different healthcare professionals say different things. I'm going crazy. In the end, you don’t trust yourself .’ Another patient replied: ‘After these experiences, it becomes difficult to trust whether it is safe to do what health professionals say. It’s best to do what you think yourself. You have to be a bad patient or friend or mother.’ (Field notes, patient education session)

The patients talked about how they feared that family or friends did not understand the rehabilitation program and thought the patients would come home cured or that the program implied that the pain was psychological. Many patients therefore feared that people believed it possible to think the pain away, implying that the patients complained and shirked their duties due to an “unreal” problem. Three of the patients talked about this in the interviews. This also emerged in a patient education session, where a patient started talking about worries concerning the next-of-kin day, where patients could invite relatives or other close ones to visit the rehabilitation unit and get information about the program:

‘They [her relatives] are here for just one and a half hours, and I’m afraid that she’ll leave here thinking that this is something you can get rid of by changing your thoughts, that you can pull yourself together, then it goes away and you’re healthy. We’re here for four weeks and get a thorough introduction, we get to ask questions. When they’re here for such a short time, they can easily go home with a simplistic conception.’ (Field notes, patient education session)

As frequently observed when patients were gathered in education sessions, the patients in this session appeared to have similar experiences. Many of them agreed with the first patient’s perception and felt strongly about it:

There’s an impassioned atmosphere in the group about this topic. The conversation moves fast. Another patient says she has decided not to invite anyone here. She has a lot of experiences showing that those closest to her don’t understand. (Field notes, patient education session)

The social worker who led this session shifted the focus by being supportive while urging the patients that they could not expect anyone to fully understand what they were experiencing:

The social worker says that you cannot make others understand to a full extent: ‘If you have held a yellow lemon in your hand and tasted it, and are going to describe how it tastes to someone who has never even touched one, you will not be able to get that person to acquire exactly the same knowledge of the lemon.’ (Field notes, patient education session)

In the patient education sessions, the professionals often acknowledged the patients’ experiences of not being believed or of not being understood regarding their pain. As in the example above, this acknowledgement could be given without directly referring to the concrete experiences of individual patients. Rather, they drew general conclusions about these phenomena while offering a possible way of challenging them.

When professionals conveyed specialised knowledge about chronic pain, it could provoke patients into disagreeing with what was said. One such instance occurred in a patient education session, when a physiotherapist described persistent pain as a false alarm, and received reactions from the audience.

The physiotherapist asks the patients what acute pain is. They suggest burns, that it is pain that passes. The physiotherapist explains that pain is an alarm signal to warn about danger. She asks the patients: ‘If chronic then?’ It is suggested that then it does not go away. The physiotherapist says that then it is a false alarm. A patient reacts to this and does not agree. The physiotherapist explains that what she means by false alarm is that with chronic pain the appropriate response is not to withdraw as in the case of acute pain. (Field notes, patient education session).

Most of the patients in our study seemed to perceive their encounters with interprofessional teams as supportive. The patients expressed their impressions of the professionals as more knowledgeable and less prejudiced than professionals elsewhere. The patients perceived the encounters as supportive in that they were seen as credible while receiving help to see their situation in a new light.

## Discussion

Our findings show that patients with chronic pain can experience a clear duality between being negatively misinterpreted outside versus positively understood and helped inside a biopsychosocial pain rehabilitation unit. In this discussion we emphasise the social meaning of the positive perceptions of encounters and how it can influence pain stigma. Regarding the patients’ perceptions of negative misinterpretations experienced elsewhere, these are consistent with earlier research showing experiences of disbelief from professionals about patients’ pain [1,5] and perceptions among family and friends of being lazy and exaggerating [37]. Such perceptions are highly relevant to this discussion, since it is a premise that patients have experiences of stigmatisation when we discuss how the positive perceptions of encounters with professionals can influence stigma. We find the negative misinterpretations to fall under the concept of stigma due to following Link and Phelan [8]; such negative beliefs are to label differences and to link a person to undesirable characteristics forming a stereotype. This can become stigma relying on the power relation. While earlier research has focussed on patient experiences of pain stigma, few studies give insight into the perceptions of being understood and helped to counter socially widespread stigmatising beliefs.

### A personal learning process

We found that patients in biopsychosocial pain rehabilitation can express positive perceptions about themselves, and about how they perceive to be seen by professionals in the unit. Earlier research has shown that patients with chronic pain can perceive themselves as strong individuals and prefer to avoid complaining about their pain [38]. Such positive self-perceptions can be empowering for gaining control of their own health [18] and for not believing in the social stigma, and thus not internalising it [3]. However, earlier research into acknowledgement for these perceptions by professionals is inconsistent. Werner and Malterud [5] found patients struggling with not being acknowledged for these perspectives by professionals. Hållstam et al. [39] found patients who finally felt understood and respected by team members in a multimodal pain rehabilitation program, though the study did not elaborate on the content of this understanding and respect. Given the increasing evidence showing interprofessional biopsychosocial pain rehabilitation programs to be the most promising treatment in relieving chronic pain, and given the lack of knowledge about the essential components and mechanisms in these programs [6,13], we suggest that acknowledgement from professionals across professions in a personal learning process is a key component.

The patient process can be viewed as a learning process due to changes in dispositions and capabilities [20] concerning views about chronic pain and reinforcement of the belief in their own ability to handle the situation. Acknowledgement from professionals can be given through both formal and informal education. An informal way is demonstrated in our findings by a meeting between a patient and an occupational therapist in which the patient initiated a discussion on overactivity, to which she had been introduced by another professional, and the occupational therapist supported and built further on the patient’s interest to learn by offering an explanation and overview of the topic. Giving information on request and spontaneously mentoring are forms of informal education [20,22]. Supporting the patient’s desire to learn is one essential aspect of facilitating learning [20]. As well as facilitating learning, these actions can acknowledge a patient for being a person with a will to change. This can counter the challenge of cultural beliefs being internalised by patients, such as the one that people with chronic pain shirk their duties or are lazy.

To diminish the internalisation of stigma in patients, one key element appears to be to clarify and help them to see life with chronic pain in a new light. Doing so can enhance the patients’ ability to reject widespread negative cultural beliefs about people with chronic pain. Clarifying misconceptions and demystifying concepts can dissolve anxiety and resistance to deviating from the previously known and embraced reality [40]. A way of helping patients to see things in a new light was demonstrated in our findings by a social worker who supported the patients’ views of being misunderstood by their close ones and offered a possible way of challenging these thoughts using a metaphor for the impossibility of making someone understand the exact experiences of others. Through a learning process with such supporting and challenging educational situations, notions about labels and stereotypes associated with chronic pain can be changed or shifted towards contrary and empowering interpretations.

Our findings show that patients can perceive professionals in interprofessional teams as being in continuity in communication across professions. Building further on what other team members have said can strengthen patient perceptions of the professionals as being in interprofessional continuity, which Hoving et al. [19] found to be related to the level of patient satisfaction with health care. Also, professionals’ explicit references to information which other professionals have received from patients, and knowing that the information has been discussed in a team, can make patients feel they are being treated in a professional and respectful manner. These kinds of actions can strengthen patients’ trust in professionals, contrary to how professionals elsewhere can be considered unreliable for giving contrary advice and for not acting in a respectful manner. This gives rise to a change in patients’ perceived relations with professionals from different professions.

For the present, we have discussed a personal learning process promoting a reduction in stigma beliefs. However, while beliefs about a group of patients is one component in the unfolding of stigma, there needs to be a relational power situation in which the less powerful can be exposed to discriminatory consequences for the stigma to occur [8].

### A transition in the patient–professional power relation

Controlling access to health care is one concrete way of having power that can result in stigmatisation, since perceptions about a patient group can entail serious discriminatory consequences [8]. When admission to pain rehabilitation is given, it can be a way of acknowledging patients as credible individuals and counteract experiences of discrimination, which one patient expressed as “being thrown around in a system” instead of receiving suitable health care.

Learning about contrary interpretations of stigma can be a process in which the relational power situation between patients and professionals can be transformed through their social encounters. We find a power transition in two different dimensions. One dimension is the empowerment of patients towards believing in their own ability to handle their situation through perceiving to be understood by professionals in ways that counter the social stigma beliefs discussed above. This can be explained as power (or capacity or empowerment) to do something, which is one part of the concept of power [41]. The extent of this transition in power depends on patients showing the right interest to learn, which can be followed up with additional informal education and acknowledgement from the professionals.

The other dimension of the transition in power can be seen through how patients in biopsychosocial pain rehabilitation perceive themselves to understand knowledge in a new “paradigm” together with the professionals, and one which outsiders do not understand. This perception places patients in a new favourable “we,” or in-group, with the professionals. We find this to be a change from the “us” and “them” situation, where people have been placed in distinct categories [8]. The patients with chronic pain can transition from being viewed as less credible by society and by professionals to being credible and knowledgeable in a group together with professionals. Admission to and support from an in-group with professionals can be viewed as a transition of power in what Reed [41] explains as having power over someone. The professionals’ power over patients becomes less striking in this process compared with the patients’ accounts of previous experiences of not being believed or of not receiving suitable health care.

Although we find a transition in the power relation, we urge an awareness of the potential challenges for patients facing a trade-off with regard to their own needs in the quest to become the good and acknowledgeable patient in order to fit into the in-group. For instance, in our findings, patient’s experiences of pain or suffering were directly discussed to a small extent only, and several statements expressed a will to avoid this kind of focus. Our findings may be due to patients trying to refute the stigma of being complainers. It can be a way of downplaying the condition, as an attempt to be normal in order to become part of the group [9,42]. This way the empowerment may also be a form of subordination without a struggle [43], which patients may not be aware of by themselves.

We find that an interprofessional biopsychosocial pain rehabilitation program can be an intervention to reduce stigma without expressing the reduction of stigma as an explicit goal. The environment in the rehabilitation unit in which patients are involved with others in a similar situation and with professionals from multiple professions can be defined as what De Ruddere and Craig [1] term an intervention strategy on the interpersonal level. There is an environment of intergroup contact where others respond in a less prejudiced manner, which enhances a personal learning process with a transition in the power relation towards diminishing stigma.

### Limitations

There are some limitations to the study, one of which is that the patients in this pain rehabilitation program where all considered to be motivated by a biopsychosocial approach as part of gaining admission to the program. Had we included people with chronic pain who were considered not to be motivated by this approach, their perceptions would differ. A large majority of the patients in the pain rehabilitation unit comprised women, and this is reflected in the sample of participants in interviews, where only one man was included. Men’s perceptions are therefore poorly represented in the study. Moreover, had we included patients who had completed the rehabilitation program and had experienced day-to-day life afterwards, we may have found their perceptions to be different.

## Conclusion

Interprofessional biopsychosocial pain rehabilitation can be an intervention to diminish internalised stigma in patients suffering from chronic pain. The study shows that patients can perceive encounters with professionals from multiple professions in pain rehabilitation as supportive. The encounters are perceived as positive due to the continuity of the professionals’ communication, while facilitating a personal learning process where patients feel they are seen as credible and helped to see their situation in a new light with contrary interpretations of social stigma beliefs. The study adds new knowledge by conceptualising how admission to rehabilitation and support from being part of an in-group with professionals in a learning process can be a transition of power in the dimensions of both “power over” and “power to.” This appears to be fundamental to diminishing pain stigma, as the occurrence of stigma is dependent on differences in power. We believe the results can be transferred to biopsychosocial pain rehabilitation settings and to other health care settings that provide treatment to patients with chronic pain, by increasing understanding of patient perceptions of encounters with professionals across professions in health care and the impact of a personal learning process on power relations and pain stigma.

## Data Availability

The data that support the findings of this study are available from Gudrun S. Battin, upon reasonable request.
